# 1003. Curbsiding Twitter: Potential Value and Patient Confidentiality Implications of Infectious Diseases Clinician Peer Consultations via Social Media

**DOI:** 10.1093/ofid/ofac492.844

**Published:** 2022-12-15

**Authors:** Ryan Boyland, Nicolas W Cortes-Penfield, Jasmine R Marcelin

**Affiliations:** University of Nebraska Medical Center, Omaha, Nebraska; University of Nebraska Medical Center, Omaha, Nebraska; University of Nebraska Medical Center, Omaha, Nebraska

## Abstract

**Background:**

Infectious Disease (ID) clinicians increasingly collaborate via social media and have adopted Twitter and the hashtag #IDTwitter for community-wide conversation. We investigated clinicians’ use of #IDTwitter for peer consultation, examining both the utility of responses and how frequently discussions risked compromising patient privacy.

**Methods:**

We reviewed English-language posts on www.twitter.com with the hashtag #IDTwitter over a 6-week period ending August 31^st^, 2021. We collected posts that we deemed examples of peer consultation, recording the post’s author, topic, engagement produced, and patient information disclosed. Finally, we recorded whether (by consensus) we judged a question meaningfully answered.

**Results:**

We identified 108 instances of peer consultation; 91 posts (84%) were consultations about a clinical scenario, 17 (16%) included a specific request for medical literature, and seven (6%) included a request for literature interpretation. Twenty-four posts (22%) contained polls, which received a mean 107 (SD 104) votes. We judged 71% of questions meaningfully answered; of these, 68% received responses including explicit justification or reasoning and 35% included citations of supporting literature. Questions were more likely to be meaningfully answered if they included polls (91.7% vs 65.5%; p=0.01). Receipt of a meaningful answer was not predicted by the questioner’s professional role and follower count, the question topic, or the post’s number of likes and shares.

A total 28/108 posts (26%) referenced the care of a specific patient, with details shared including gender (74%), age (26%), radiographic or clinical images (7%), and other potentially identifying data (7%). Patients were stated or implied to be currently under the clinician’s care in 70% of cases, and 61 clinicians (92.4%) had their employer listed in their profile or immediately available via internet search.

**Conclusion:**

ID clinicians’ peer consultations via #IDTwitter appeared frequently useful, particularly when questions were accompanied by polls. However, most participants sharing patient data were readily identifiable, and best practices to limit sharing of nonessential data and safeguard patient privacy are needed.

**Disclosures:**

**Jasmine R. Marcelin, MD**, Pfizer (Grant reviewer): Honoraria.

